# Is Parkinson's Disease Truly a Prion-Like Disorder? An Appraisal of Current Evidence

**DOI:** 10.1155/2015/345285

**Published:** 2015-01-13

**Authors:** Aneesha Chauhan, Alexander F. Jeans

**Affiliations:** ^1^Hertford College, University of Oxford, Catte Street, Oxford OX1 3BW, UK; ^2^Department of Pharmacology, University of Oxford, Mansfield Road, Oxford OX1 3QT, UK

## Abstract

Parkinson's disease (PD) is the world's second most common neurodegenerative disease and most common movement disorder. Characterised by a loss of dopaminergic neurons and the development of intraneuronal inclusions known as Lewy bodies, it has classically been thought of as a cell-autonomous disease. However, in 2008, two groups reported the startling observation of Lewy bodies within embryonic neuronal grafts transplanted into PD patients little more than a decade previously, suggesting that PD pathology can be propagated to neighbouring cells and calling basic assumptions of our understanding of the disease into question. Subsequent research has largely served to confirm this interpretation, pointing towards a prion-like intercellular transfer of misfolded *α*-synuclein, the main component of Lewy bodies, as central to PD. This shift in thinking offers a revolutionary approach to PD treatment, potentially enabling a transition from purely symptomatic therapy to direct targeting of the pathology that drives disease progression. In this short review, we appraise current experimental support for PD as a prion-like disease, whilst highlighting areas of controversy or inconsistency which must be resolved. We also offer a brief discussion of the therapeutic implications of these discoveries.

## 1. Introduction

First described as “the shaking palsy” in 1817, Parkinson's disease (PD) is the second most common neurodegenerative disease, prevalent in 1% of the population over the age of 60 [1]. The unprecedented rate of world population ageing is predicted to dramatically increase the number of PD sufferers, reducing overall quality of life and increasing healthcare burden [2]. In the late 1980s and early 1990s, the search for better treatment strategies prompted trials of embryonic neuronal transplants [3]. When, some years later, these patients came to autopsy, a startling observation was made: PD pathology, in the form of Lewy bodies and neurites, was now present in the grafts, raising the possibility of spread from diseased tissue to the young, transplanted neurons [4–6]. This unexpected discovery engendered a novel field of research that is leading to a radical shift in understanding. This review focuses on the evidence examining whether, contrary to classical thinking, PD does indeed progress through non-cell-autonomous mechanisms. The earliest hypotheses concerning these novel mechanisms suggested a prion-like mode of disease propagation, with misfolded *α*-synuclein (*α*syn, the main component of Lewy pathology), the leading candidate for the transmissible protein agent [7]. As we will discuss, subsequent work has offered strong support for this model, leading to a novel view of PD pathogenesis and progression with exciting therapeutic implications, since targeting *α*syn spread could lead to new therapies that prevent, halt, or slow PD progression, thereby alleviating both motor and nonmotor symptoms of PD.

## 2. PD Pathology

The traditional understanding of PD as a movement disorder centres on dopaminergic neuronal loss in the substantia nigra pars compacta (SNPC) [8], as typically demonstrated by a macroscopic reduction in neuromelanin pigmentation and microscopically confirmed by decreased immunoreactivity for dopaminergic neuronal markers: tyrosine hydroxylase (TH, an essential enzyme in dopamine synthesis) and dopamine transporter (DAT, responsible for dopamine reuptake and recycling) [9]. The loss of these neurons causes much of the characteristic motor disturbance that gives rise to the cardinal clinical signs of PD: bradykinesia, postural instability, muscle rigidity, and tremor [8]. Accordingly, most current therapies aim to restore motor function by raising dopamine levels within the remaining functioning dopaminergic neurons, thus boosting their effectiveness.

Intraneuronal inclusions, known as Lewy bodies or Lewy neurites, are also present and are considered the hallmark of microscopic PD pathology. The main component of these abnormal aggregates was an enigma until it was discovered that monoclonal antibodies against *α*syn, a presynaptic protein suggested to regulate neurotransmission, intensely labelled Lewy bodies [10]. Both mutations and duplication or triplication of the *α*syn gene cause an autosomal dominant form of PD [11], strongly implicating *α*syn in PD pathogenesis. The *α*syn present in Lewy pathology exhibits a conformational change from the native soluble protein to an insoluble, fibrillar form. These fibrils are rich in *β*-pleated sheets, shown clearly by intense staining with thioflavin S. Over 90% of this *α*syn, compared to 5% normally, is phosphorylated at serine-129 (pser-129), which promotes fibril formation [12]. Pathological human *α*syn shows ubiquitin immunoreactivity [13], indicating that at least some of it is targeted for degradation by the proteasome; however, the degree of ubiquitination is variable even within individual cases [14], which may reflect the fact that other processes have also been implicated in the degradation of *α*syn, most notably the autophagy-lysosomal pathway [15].

## 3. Prion Diseases

**Figure 1:**
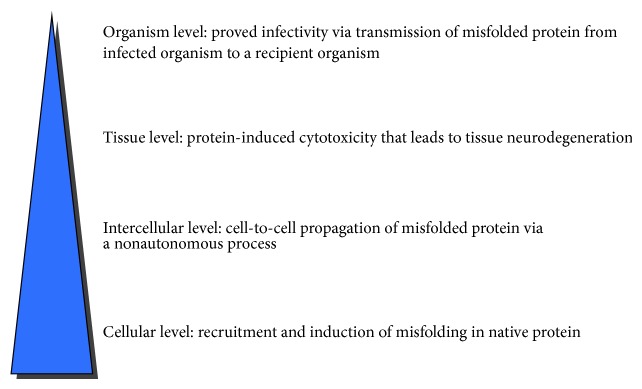
Diagram showing the criteria that must be satisfied for a disease to qualify as a prionopathy [19]. The most unique attribute of prion diseases is their transmissibility between individuals via transfer of pathological protein alone.

The science of infectious disease was revolutionised in 1982 when Prusiner postulated that proteinaceous infectious particles (prions) devoid of nucleic acids cause the disease scrapie in animals [16]. This once-heretical theory is now generally well accepted due to vast experimental support [17]. Composed of misfolded forms of native cellular proteins, prions are capable of perpetuating infection by catalysing the conversion of their normally folded counterparts into the misfolded conformation. In humans, all known prionopathies are neurodegenerative and are caused by misfolded prion protein (PrP) [18]. Protein misfolding is a common theme in most of the major neurodegenerative diseases, and mechanistic similarities to prionopathies have been suggested for a number of these, although PD has attracted the most interest from this perspective. In order for a disease to qualify as a prionopathy, specific criteria at both microscopic and macroscopic levels have been proposed [19], which are shown in Figure 1.

Currently, there is no evidence of interorganismal infectivity in PD; therefore it can not* a priori* be a true prion disease according to this definition [20]. Despite this, a consideration of the other criteria in light of our mechanistic understanding of PD and other neurodegenerative diseases suggests that the line between infectious disease and what is traditionally thought of as cell-autonomous neurodegeneration is much less clear than originally thought.

## 4. The Transplant Studies

Dopamine replacement therapy, the mainstay of current treatment for PD, can demonstrate limited efficacy and may be further complicated by drug-induced dyskinesias. Furthermore, PD leads to many nonmotor symptoms, such as autonomic dysfunction and cognitive and mood disturbances, all of which respond very poorly to dopamine replacement. These problems emphasise the need to target the cause of the progressive neuronal loss [21]. Consequently, cell replacement therapy was considered to be a logical treatment strategy for PD.

In a series of open-label trials in the 1990s [3], fetal mesencephalic dopaminergic neuronal grafts into the putamen, or the putamen and caudate nucleus of PD patients, were reported to induce long-term functional benefits. However, the questionable sensitivity of subjective outcome measures to placebo effects engendered two new double-blind trials which, unfortunately, did not find such benefits, suggesting placebo effects, and/or observer bias affected earlier trials [22, 23]. Nevertheless, in 2008 results from the postmortem analyses of 9 patients who died between 11 and 16 years after graft insertion were published. Incredibly, pser-129-*α*syn-positive Lewy-like inclusions were observed within the mesencephalic grafts of 4 patients [4–6], a completely uncharacteristic finding in such young neurons. Reduced DAT and TH levels were also seen, indicating graft dysfunction. It was suggested that the appearance of pathology was time-dependent due to a lack of pathology in grafts younger than 4 years old and an increased percentage of *α*syn-positive dopaminergic neurons in a 16-year-old graft compared to a 12-year-old graft from the same person [6].

In light of these observations, a hypothesis that PD was a transmissible disease with prion-like features was proposed [6]. However, Lewy pathology was not detected in all grafts [24], casting doubt upon the theory of host-to-graft transmission. Further studies were required to understand what was occurring. Over the subsequent six years, a substantial body of work has amassed dedicated to examining the prion-like hypothesis for PD. Below, this evidence is summarised with regard to the specific cellular, intercellular, and tissue level criteria for prionopathies introduced above [19]. As the remaining criterion, for interorganismal infectivity, has not been reported in PD, the term “prion-like” must strictly be used. It might reasonably be argued, however, that demonstrated host-graft spread might go at least some way towards meeting this requirement.

## 5. Cellular Level

An important characteristic of prion disease is that a pool of endogenous, natively folded PrP (PrPc) is required as a substrate for conversion by, and to, the pathological, misfolded form (PrPsc) [25]. Via the use of *α*syn knockout tissues and animals, endogenous *α*syn has been confirmed as similarly necessary for the development of *α*syn pathology both* in vitro* [26] and* in vivo* [27]. Furthermore, the pathological forms of both *α*syn and PrP are structurally similar, being rich in *β*-pleated sheets [28], prone to fibril formation, and resistant to normally denaturing agents.

**Figure 2:**
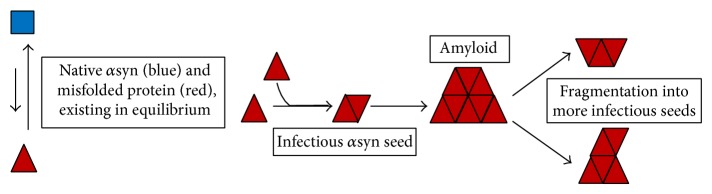
The seeding hypothesis of prion disease pathogenesis, adapted from [25].

There are two mainstream theories regarding the mechanism of conversion of PrP (PrPc) to the misfolded “scrapie” form (PrPsc): the refolding hypothesis and the currently more favoured seeding hypothesis [25] (Figure 2). There is evidence of *α*syn seeding in Lewy body formation, which has best been demonstrated* in vitro* [28].

One study added myc-tagged recombinant *α*syn preformed fibrils (PFFs) to cells overexpressing *α*syn, leading to the formation of Lewy-body-like inclusions. Anti-myc antibodies only labeled the centres of inclusions; endogenous *α*syn had been recruited and converted to form the periphery [29]. Whilst this demonstrates seeding, nonphysiological lipofection agents were used to optimise intracellular delivery of PFFs. However, other studies have demonstrated seeding in immunofluorescence studies without lipofection agents. In one, cells expressing either green or red fluorescence-labeled *α*syn were cocultured, generating inclusions with a central core of green-labeled *α*syn surrounded by endogenous red-labeled *α*syn [30]. A more recent example used a methodology for the synthesis of artificial prions to create short *α*syn amyloid fibrils; a single exposure to these was sufficient to induce aggregation of endogenous *α*syn in human neuroblastoma SH-SY5Y cells [31].

These similarities between prionopathies and PD on a molecular and cellular level would seem to create a stable foundation for the prion-like hypothesis.

## 6. Intercellular Level

The spread of Lewy pathology in graft patients is likely to be due to intercellular transfer of *α*syn. There are numerous mechanisms by which this could occur, from cellular release and uptake of *α*syn to direct transmission via nanotubes [7].

### 6.1. *α*syn Secretion and Uptake

Despite a lack of secretory signal peptide sequence, *α*syn is present in the cerebrospinal fluid of individuals with or without PD [32]. There is support for exocytosis-mediated *α*syn release* in vitro*, shown to be independent of cell death or membrane leakage. This secretion is reduced by low temperature, which also slows vesicular exocytosis, but is unaffected by Brefeldin A, which inhibits conventional exocytosis. These data suggest that *α*syn is secreted via an unconventional vesicular pathway [33], although current opinion on what form this could take is divided. Electron microscopy shows that *α*syn can be found within large dense-core vesicles [33]. Alternatively, it has been suggested that exosomes, small membrane vesicles released into the extracellular space, are the carrier and that exosome-associated *α*syn is more likely to be internalised than free *α*syn [34]. Thus, it is possible that there may be more than one *α*syn secretory pathway.

The need for lipofection agents in many experiments has highlighted the difficulty in demonstrating meaningful *α*syn internalization [29], although unaided uptake both* in vitro* and* in vivo* has been demonstrated by Desplats and colleagues [35]. Fluorescently labeled mouse cortical neuronal stem cells (MCNSCs) were injected into the hippocampus of transgenic mice expressing human *α*syn under the control of the brain-specific Thy-1 promoter. Four weeks later, 15% of grafted cells displayed human-*α*syn immunoreactivity. Some cells developed *α*syn-positive inclusions although, unexpectedly, fibrillar *α*syn was not detected. This pathology was not observed in grafts inserted into nontransgenic mice: *α*syn is therefore essential for host-to-graft transmission. However, the hippocampus is not a site relevant to grafts in clinical PD treatment, and MCNSCs are proliferative, unlike most neurons. Accordingly, Hansen and colleagues transplanted wild-type mouse embryonic mesencephalic neurons into the striatum of transgenic mice overexpressing human-*α*syn under the control of the native mouse *α*syn promoter. Six months later, 5% of transplanted neurons were human-*α*syn-positive [30].

Both Desplats and Hansen give evidence for an endocytic mechanism of *α*syn uptake via the use of inhibitors [30, 35]. Other studies find that this is true for aggregated forms of *α*syn but that monomeric *α*syn can be directly translocated across the plasma membrane, suggesting that at least two internalisation pathways may exist which are *α*syn state-dependent [36]. However, one notable disparity between the Desplats and Hansen studies is the proportion of cells displaying *α*syn immunoreactivity (15% in 4 weeks versus 5% in 6 months). This is likely due to differences in experimental design, which include different promoters driving *α*syn expression, the types of transplanted cell utilized, and differing brain areas studied. Furthermore, Hansen and colleagues did not observe seeding after uptake* in vivo*, and a similar study in rats also reported little aggregation [37]. Despite these variations in experimental observations, we believe there is overall strong evidence for *α*syn secretion and uptake.

### 6.2. Tunneling Nanotubes (TNTs)

TNTs are a recently discovered form of direct cell-to-cell communication. They are F-actin containing tubes with diameters of less than 200 nm that connect the cytoplasm of two cells. Intercellular transfer of PrPsc [38] has been confirmed and, recently, *α*syn has been found within TNTs between glioblastoma cells [39]. This preliminary evidence will require further investigation to elucidate the role, if any, of TNTs in *α*syn transmission in PD.

### 6.3. Intercellular Transfer: The Braak Hypothesis

The Braak hypothesis is perhaps the currently best-accepted model of PD progression and would be highly consistent with a prion-like mechanism of spread. Derived from the postmortem analyses of 100 *α*syn-positive subjects, it suggests that, in PD, Lewy pathology affects regions in the brain in a stereotypic, topographical manner. Lewy pathology severity is also suggested to correlate with the clinical progression of PD symptoms [40]. Braak suggests that the stereotypical propagation of pathology depends partly on the vulnerability of specific neuron types. A potential explanation of the particular vulnerability of SNPC dopaminergic neurons might be furnished by the suggestion that oxidative stress and *α*syn aggregation form a positive feedback loop [28], coupled with evidence that these neurons are particularly susceptible to oxidative stress, due to both metabolic demands particular to this population and dopamine oxidation with generation of reactive oxidative species [41]. Dopamine is also known to induce the formation of soluble toxic aggregates of *α*syn, which have been shown to be capable of replication by self-propagation* in vitro* [42].

**Figure 3:**
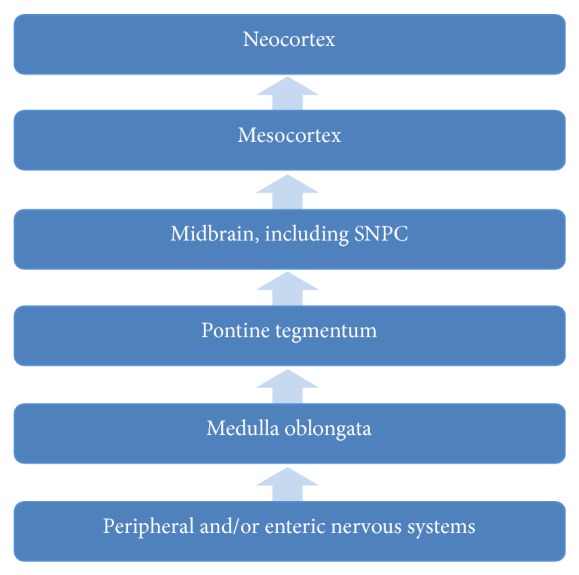
The 6 stages of PD according to the Braak hypothesis. Pathology begins in the enteric nervous system and progresses to the neocortex.

The Braak hypothesis proposes that *α*syn pathology begins, due to environmental insult, within enteric epithelium. From here, it travels retrogradely via the axons of enteric nervous system (ENS) neurons to their somata within the intermediolateral column of the spinal cord and then to the dorsal motor nucleus of the vagus (DMNV) in the medulla. Thereafter, it is able to reach the pons and substantia nigra and, beyond that, vulnerable regions of the cortex [7] (Figure 3). There is some experimental support for this scheme: in rats, intragastric administration of rotenone, a mitochondrial complex I inhibitor that generates Lewy-like inclusions, generated Lewy pathology in the enteric nervous system (ENS) which spreads to the substantia nigra in agreement with the Braak hypothesis [43]. The pathology only appeared in synaptically connected areas, suggestive of transsynaptic transmission, and a later study using the same model showed that transection of autonomic axons was sufficient to halt the spread of enteric *α*syn pathology [44]. However, the Braak hypothesis remains controversial, and challenges exist for this particular account of pathological *α*syn spread, as well as for interneuronal spread more generally. These include several studies that find distributions of *α*syn pathology in PD that would not be predicted by the stereotyped Braak pattern of disease spread and the observation that *α*syn pathology is also the predominant histopathological characteristic of other diseases, most notably multiple system atrophy (MSA), which feature a completely different pattern of CNS involvement and spread than PD [7]. There is also the related observation that some forms of substantia nigra degeneration and clinical parkinsonism exhibit no Lewy pathology at all [45].

Most of the specific conflicts with the Braak hypothesis can be resolved by extending the latter to include the possibility of anterograde spread, which is still quite compatible with a prion-like model, which greatly broadens the possible patterns and sequences of involvement one could see [46]. Many of the remaining inconsistencies can be addressed by considering the role of deposited *α*syn: is it a reliable marker of cell damage, therefore indicating the degree to which regions are affected by the disease, or is it part of a protective response which mitigates cellular damage? In the latter case, Lewy bodies might mark those neurons resisting degeneration, which might actually be most active elsewhere [7]. If the latter were true even some of the time, then the assumption of so many studies, that *α*syn pathology correlates with degeneration, could be seriously flawed, with clear implications for study conclusions. In support of this idea, recent work examining the relationship between nigral cell loss, the duration of motor symptoms, and the distribution and density of Lewy pathology found no correlation [47]. It has also been shown that Lewy bodies are absent from the majority of cells showing apoptotic changes [48].

An imperfect correlation of pathological *α*syn deposits with active disease may simply reflect the emerging theory that it is not visibly aggregated *α*syn, but oligomeric *α*syn, that is cytotoxic [49], a theory that has also found support in prion diseases [50]. In keeping with this, it has been shown that stabilisation of *α*syn in oligomeric form increases cytotoxicity, whilst reduced oligomer formation decreases cytotoxicity [51].

## 7. Tissue Level

In prionopathies, the protein itself is toxic and leads to rapid neurodegeneration [18]. The prion-like hypothesis of PD and the Braak hypothesis both propose that *α*syn can serve the same function. In one study, PFFs were injected intrastriatally into nontransgenic mice and *α*syn spread to the dopaminergic cells of the SNPC was observed by 30 days of postinjection (dpi), with negligible cell loss. However, by 100 dpi, the total number of cells and striatal dopamine concentration had decreased, suggesting that *α*syn Lewy pathology is cytotoxic [52]. This cell death correlated with declines in motor function.* In vitro*, *α*syn has been shown to lead to caspase-3 activation [35]. Cell-cell spread in the opposite direction has also been elegantly demonstrated via the experimental induction of human-*α*syn overexpression in the right substantia nigra of rats, along with bilateral striatal transplant of embryonic dopaminergic neurons. This led to *α*syn immunoreactivity in the right striatal transplant but not the left [53]. Furthermore, different sites of injection of pathological *α*syn-containing material elicited different and connectivity-dependent patterns of *α*syn spread, although it must be noted that nonphysiologically high concentrations of *α*syn were used to elicit widespread transfer in these experiments [52].

Overall, the evidence provides substantial support for prion-like transfer of *α*syn, but controversies remain; in particular, one of the most prominent models for PD propagation, the Braak hypothesis, which would be entirely consistent with prion-like spread, has not been clearly proved. To properly address this question experimentally, a technique to longitudinally monitor *α*syn spread will need to be developed, which will also allow prion-like transfer to be examined over time.

## 8. Therapeutic Strategies

The field of research into prion-like *α*syn spread is very young; however, the therapeutic implications could be huge. Inhibition of *α*syn transmission could offer a disease-modifying approach with the potential to prevent both motor and nonmotor decline in patients.

**Figure 4:**
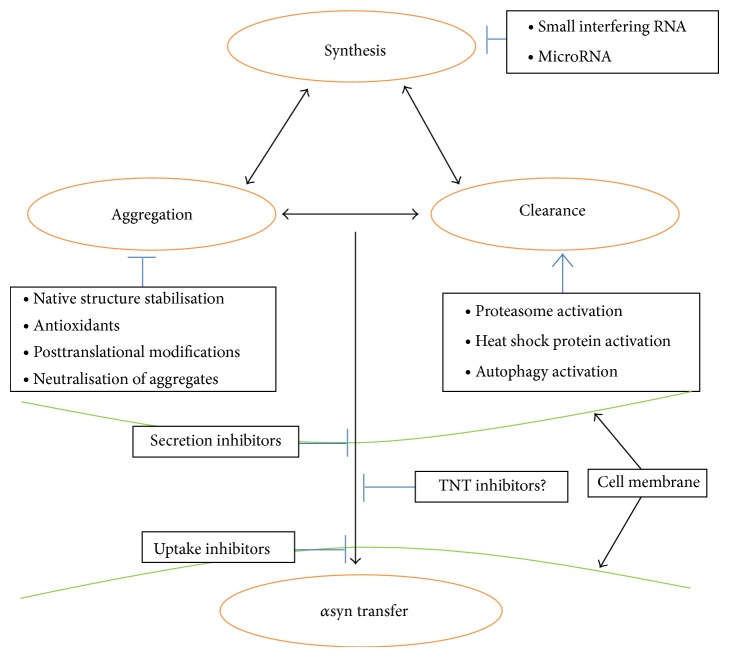
Possible therapeutic strategies to decrease *α*syn transmission in PD. This is not an exhaustive representation; see [7, 57, 59].

A summary of possible treatment strategies targeting *α*syn transmission is represented in Figure 4. Whilst many of these remain theoretical, some have been trialled* in vitro* for PD or other protein misfolding diseases. One possibility is to increase the resistance of *α*syn to seeding. In transthyretin amyloidosis, where transthyretin misfolds to cause disease, compounds have been developed to stabilise the protein as a functional tetramer [54]. As *α*syn can exist as a folded tetramer that is resistant to aggregation [55], this strategy could be adopted for PD treatment. Another attractive strategy is to decrease the amount of *α*syn available for misfolding, which may cause only few side effects since the protein shows a high degree of cellular redundancy in mammals. One study reported that interfering RNA against *α*syn conferred resistance to MPP+ exposure, which usually causes PD-like degeneration [56]. Such strategies would be of particular interest due to their ability to halt the disease at the very start of the pathological cascade. Other strategies could directly or indirectly reduce the rate of intercellular transfer; while the mechanisms underlying this are currently not entirely clear, once they are better understood it is hoped that specific inhibitors of the relevant processes can be designed or may even exist already. Finally, neuronal transplants could still be useful if the benefits previously seen in clinical trials could be maximised, whilst simultaneously minimising graft dysfunction. An ongoing multicentre trial, TRANSEURO, aims to elucidate the factors affecting transplant efficacy to improve such cell replacement therapies [57].

## 9. Future Studies and Conclusions

While many studies have provided much good evidence for prion-like *α*syn transfer, progress is hampered by many of the limitations of current experimental animal models, which are an imperfect replication of human PD. Of course, such problems are far from unique to this field. While many improvements would be desirable to ensure greater applicability to human disease, perhaps the most significant shortcomings are the chronicity of the studies, which take place in fairly short-lived organisms, and the nonphysiological levels of *α*syn protein which have been used to elicit spread, both of which are clear confounds when considering a process which takes place over many years and with native levels of *α*syn in PD. With regard to the latter problem, it is noteworthy that while the majority of studies use cells or animals overexpressing *α*syn at high levels, this is not always necessary for PD pathology to develop if neurons are exposed to PFFs [26].* In vitro* models also have an important role to play in allowing high throughput approaches and a level of access that* in vivo* models often cannot support. To this end, the use of human induced pluripotent stem cells is an exciting development which promises all the advantages of the* in vitro* approach coupled with the unprecedented ability to study dynamic disease processes in real time in living human neurons differentiated along the lineages of relevant populations such as SNPC dopaminergic neurons [58].

In summary, the role of prion-like spread in PD progression remains an exciting area of research. Whilst there are still unanswered questions, many studies corroborate the idea that *α*syn spread via a prion-like infectious process is central to PD. The therapeutic implications of this conclusion are powerful indeed and have the potential to revolutionise treatment of both motor and nonmotor manifestations of this devastating disease.
